# Patient Blood Management after Hematopoietic Stem Cell Transplantation in a Pediatric Setting: Starting Low and Going Lower

**DOI:** 10.3390/diagnostics13132257

**Published:** 2023-07-03

**Authors:** Claudia Del Fante, Cristina Mortellaro, Santina Recupero, Giovanna Giorgiani, Annalisa Agostini, Arianna Panigari, Cesare Perotti, Marco Zecca

**Affiliations:** 1 Immunohaematology and Transfusion Service, Fondazione IRCCS Policlinico San Matteo, 27100 Pavia, Italy; c.mortellaro@smatteo.pv.it (C.M.); c.perotti@smatteo.pv.it (C.P.); 2 Pediatric Hematology/Oncology, Fondazione IRCCS Policlinico San Matteo, 27100 Pavia, Italy; s.recupero@smatteo.pv.it (S.R.); g.giorgiani@smatteo.pv.it (G.G.); a.agostini@smatteo.pv.it (A.A.); a.panigari@smatteo.pv.it (A.P.); m.zecca@smatteo.pv.it (M.Z.)

**Keywords:** patient blood management, hematopoietic stem cell transplantation, transfusions, children

## Abstract

Despite the substantial transfusion requirements, there are few studies on the optimal transfusion strategy in pediatric patients undergoing hematopoietic stem cell transplantation (HSCT). Our study aimed to retrospectively analyze red blood cell (RBC) and platelet (PLT) transfusion practices during the first 100 days after HSCT at the pediatric hematology/oncology unit of our hospital between 2016 and 2019, due to a more restrictive approach adopted after 2016. We also evaluated the impact on patient outcomes. A total of 146 consecutive HSCT patients were analyzed. In patients without hemorrhagic complications, the Hb threshold for RBC transfusions decreased significantly from 2016 to 2017 (from 7.8 g/dL to 7.3 g/dL; *p* = 0.010), whereas it remained the same in 2017, 2018, and 2019 (7.3, 7.2, and 7.2 g/dL, respectively). Similarly, the PLT threshold decreased significantly from 2016 to 2017 (from 18,000 to 16,000/μL; *p* = 0.026) and further decreased in 2019 (15,000/μL). In patients without severe hemorrhagic complications, the number of RBC and PLT transfusions remained very low over time. No increase in 100-day and 180-day non-relapse mortality or adverse events was observed during the study period. No patient died due to hemorrhagic complications. Our preliminary observations support robust studies enrolling HSCT patients in patient blood management programs.

## 1. Introduction

The transfusion of blood products is lifesaving in the presence of severe anemia or bleeding, and it is essential to support aplasia during bone marrow reconstitution after hematopoietic stem cell transplantation (HSCT) [1,2]. However, blood resources are limited and costly and are not risk-free, as some transfusion-transmitted infections and/or adverse transfusion reactions (acute and late) may occur, despite the high safety of blood products [3,4,5]. In order to optimize the transfusion strategy, during the last decades several studies have been conducted in many different medical fields (i.e., surgery) introducing the concept of patient blood management (PBM) [6]. PBM is a multidisciplinary evidence-based concept aimed at optimizing patient hemoglobin (Hb) concentration, minimizing blood loss, and improving hemostasis to increase patient safety [7]. To date, many studies have been conducted so far to implement PBM in different adult contexts; conversely, few studies are available in pediatric settings [8,9,10,11,12,13,14]. Likewise, adverse events related to blood transfusion in children are not fully understood; in fact, age (i.e., neonates or infants), genetic susceptibility, disease, etc., may play a role in the development of adverse reactions [15]. In addition, high transfusion requirements also increase the risk of immune-mediated reactions and iron overload in pediatric patients undergoing HSCT [16,17]. Results from several studies conducted in surgical and critically ill pediatric patients have shown that a restrictive Hb cut-off (7 g/dL) is a valid alternative to a more liberal strategy (Hb higher than 8 g/dL, based on clinician’s discretion) with no increase in an adverse outcome [18,19,20,21,22]. Moreover, a hemoglobin threshold of 7 g/dL demonstrated a benefit (reduction of the transfusion number with no negative impact on clinical parameters, including mortality) compared to that of 9.5 g/dL in a randomized trial conducted in children admitted to a pediatric intensive care unit [23,24]. The same issue regarding the transfusion of RBC can be extended to both prophylactic or therapeutic platelets (PLT) and plasma transfusions, which also appear to be overused. Very few studies are available on transfusion strategy in pediatric patients undergoing HSCT, even though these patients require substantial transfusion support [25,26,27]. The lack of sufficient evidence resulting from the available studies does not allow for making definitive recommendations on pre-transfusion Hb and PLT thresholds in this setting [28].

Since 2017, at the pediatric hematology/oncology unit of our hospital a progressively more restrictive approach has been adopted by the attending physicians, given the demonstration of the benefit of a restrictive transfusion policy (Hb threshold 7 g/dL) reported in the available studies and also in critically ill children [23,24], as well as the lack of specific national transfusion guidelines in pediatric HSCT patients. In fact, the internal transfusion strategy in place prior to 2017 provided for routine RBC and PLT transfusions in HSCT patients when Hb and PLT counts were 8 g/dL and 20,000/µL, respectively.

Our study retrospectively analyzed the RBC and PLT transfusion practices adopted for children and adolescents undergoing HSCT at the pediatric hematology/oncology unit of our hospital between 2016 and 2019 to highlight the differences in transfusion policy (more liberal or more restrictive) between the 2016 and 2017–2019 periods. In fact, the change in transfusion thresholds occurred in 2017, with a reduction from 8 to 7.5 g/dL for RBC and from 20,000 to 15,000/μL for PLT in stable patients without major hemorrhagic complications. The primary objective of our study was to verify the feasibility of a more restrictive transfusion policy also in a pediatric population and the adherence of the physicians to the new transfusion thresholds. Secondary endpoints were to evaluate the effect of the change in the transfusion policy on the number of RBC and PLT transfusions administered to the patient and on survival and non-relapse mortality in the first 6 months after HSCT. Our analysis also compared clinical and laboratory parameters to assess the impact of the different transfusion policies on patients’ outcomes.

## 2. Materials and Methods

The study was conducted in accordance with the Declaration of Helsinki and approved by the local Institutional Review Board of Fondazione IRCCS Policlinico San Matteo Pavia (Protocol 15410/14). Written informed consent was obtained from patients and guardians, according to the Declaration of Helsinki. Data were collected retrospectively by accessing the dedicated computer programs of the blood bank and clinical laboratory analysis of our hospital as well as the electronic patient record. Data of all consecutive pediatric HSCT recipients (both allogeneic and autologous) regarding the first 100 days post-transplantation were collected during the period from the 1 January 2016 to 31 December 2019 timeframe. Overall, prior to the change in transfusion policy, the internal protocol provided for RBC transfusion for both stable, uncomplicated, inpatients, and outpatients when Hb values were around 8 g/dL and PLT around 20,000/µL. After January 2017, the threshold for RBC transfusions was lowered to <7.5 g/dL and that of PLT transfusions to <15,000/µL. However, there could be exceptions based on treating physician considerations. Unstable patients (e.g., those with severe mucositis, gastrointestinal acute GVHD, hemorrhagic cystitis) could be transfused with higher thresholds depending on the clinical condition.

Collected data included demographics, underlying disease, conditioning regimen, stem cell source, date and type of transplant, complications (GvHD, infectious diseases, etc.), time to engraftment, 6 months survival probability post-transplantation (see Table 1). Hb and PLT values obtained from the clinical laboratory database immediately before each transfusion (pre-transfusion) and within 24 h after each transfusion (post-transfusion) as well as type of component (RBC or PLT), number, and date of transfusion were recorded. Patients’ records during each admission were also analyzed. Of note, it was not possible to extract from the database the precise volume of RBC transfusion administered.

The blood bank protocol required that all patients undergoing RBC transfusion received irradiated/leukoreduced RBC with the same AB0-Rh/Kell phenotype whenever possible. If not, the best match for the individual patient was released. In addition, irradiated leukodepleted apheresis PLT, resuspended in an additive solution, regardless of the patient’s AB0 blood group, were administered to minimize any possible risk related to white blood cell infusion and to improve pH and PLT quality, as per internal policy. When apheresis PLT were not available, irradiated pooled PLT were administered.

Any further decision considered for the single patient during the entire study period was taken at the discretion of the attending physician.

### Statistical Analysis

Patient demographics and transplant-related variables (diagnosis, stem cell source, donor type, conditioning regimen, and GvHD prophylaxis) were analyzed using descriptive statistics. Comparative analysis of categorical variables was performed using the unpaired *t*-test or Fisher’s exact test, whereas the Wilcoxon rank-sum test was used for continuous variables. Kaplan–Meier curves were used to describe survival (SUR) and event-free survival (EFS), and the log-rank test was used for comparisons. Non-relapse mortality (NRM) was expressed as cumulative incidence, as were acute and chronic GvHD, neutrophil and platelet engraftment, and graft failure; comparisons were made using Gary’s test. Results were expressed as median, 25th and 75th percentiles and range, or as percentages and 95% confidence intervals (95% CI), as appropriate. Two-sided *p* values < 0.05 were considered statistically significant. All *p* values were reported in detail.

Analyses were performed with the statistical software NCSS 10 (NCSS, LLC. Kaysville, UT, USA) and STATA 15 (StataCorp. 4905 Lakeway Drive, College Station, TX 77845, USA).

## 3. Results

During the four-year study period, a total of 146 consecutive pediatric patients were analyzed (83 males, 63 females). The median age at transplantation was 8 years (range, 0.6–18 years), and the median body weight at transplantation was 24 kg (range, 6–90). Seventy-seven patients (53%) had malignant disease and sixty-nine (47%) had non-malignant hematologic disorders. In addition, 13 patients (9%) received an autologous HSCT, and 133 (91%) received an allogeneic HSCT from a matched family donor in 27 (19%), a matched unrelated donor in 50 (34%), and a haploidentical family donor in 56 (38%). All patients underwent myeloablative conditioning. Further details on patient and graft characteristics are shown in Table 1. The relative distribution of patient demographics and transplant procedure characteristics remained comparable throughout the 4-year study period.

Table 2 summarizes the clinical outcomes of the patients enrolled in the study. One hundred and twenty-one patients (83%) were alive after HSCT, and the median observation time for surviving patients was 3.2 years (range, 1–7.2 years). Twenty-five patients (17%) died at a median of 3.5 months after HSCT (range, 7 days–2.8 years). The cause of death was disease progression in 11 patients (7%), while 14 patients (10%) died in remission due to transplant-related causes. Acute GVHD was the primary cause of death in 3 patients, and 5 patients died due to infectious complications, 4 patients died due to multiple organ failure, 1 patient died due to macrophage activation syndrome (MAS), and 1 patient died due to veno-occlusive disease (VOD). No patient died from hemorrhagic complications.

Table 3 shows pre-transfusion Hb levels, and the number of RBC transfusions administered during the first 100 days after transplantation for the entire study population and for two subgroups of patients, those without hemorrhagic transplant complications and those who developed hemorrhagic transplant complications (i.e., severe hemorrhagic cystitis due to Polyomavirus BK or Adenovirus infection, severe acute gastrointestinal GVHD, severe gastrointestinal thrombotic mycroangiopathy). In patients without hemorrhagic complications, the Hb threshold for RBC transfusions decreased significantly from 2016 to 2017 (from 7.8 g/dL to 7.3 g/dL; *p* = 0.010) and remained at the same low level in 2017, 2018, and 2019 (7.3, 7.2, and 7.2 g/dL, respectively). On the contrary, as expected, in the group of 8 patients who developed hemorrhagic transplant complications, the pre-transfusion Hb level remained unchanged over the 4-year study period based on clinical decisions.

As shown in Table 4, in children without hemorrhagic transplant complications, the number of RBC transfusions given during the first 100 days after HSCT was always very low (median = 3 RBC transfusions; range, 0–12) and remained consistently low over time, whereas, as expected, it remained significantly higher in patients with hemorrhagic transplant complications (median = 18 RBC transfusions per patient; range, 13–82. *p* < 0.01).

Table 5 shows the pre-transfusion PLT levels and the number of PLT transfusions during the first 100 days after transplantation for the entire study population and for two subgroups, those with and without hemorrhagic complications. The median pre-transfusion PLT count decreased from 18,000/μL in 2016 to 15,000/μL in the following years (*p* = 0.026) in patients without hemorrhagic transplant complications, while it remained higher and unchanged in patients with hemorrhagic transplant complications.

The number of PLT transfusions in patients without hemorrhagic transplant complications remained low throughout the study period (median 6 PLT transfusions per patient; range, 0–33), while, as expected, it was significantly higher in patients with hemorrhagic transplant complications (median 35; range, 11–63; *p* < 0.01)—Table 6.

**Figure 1:**
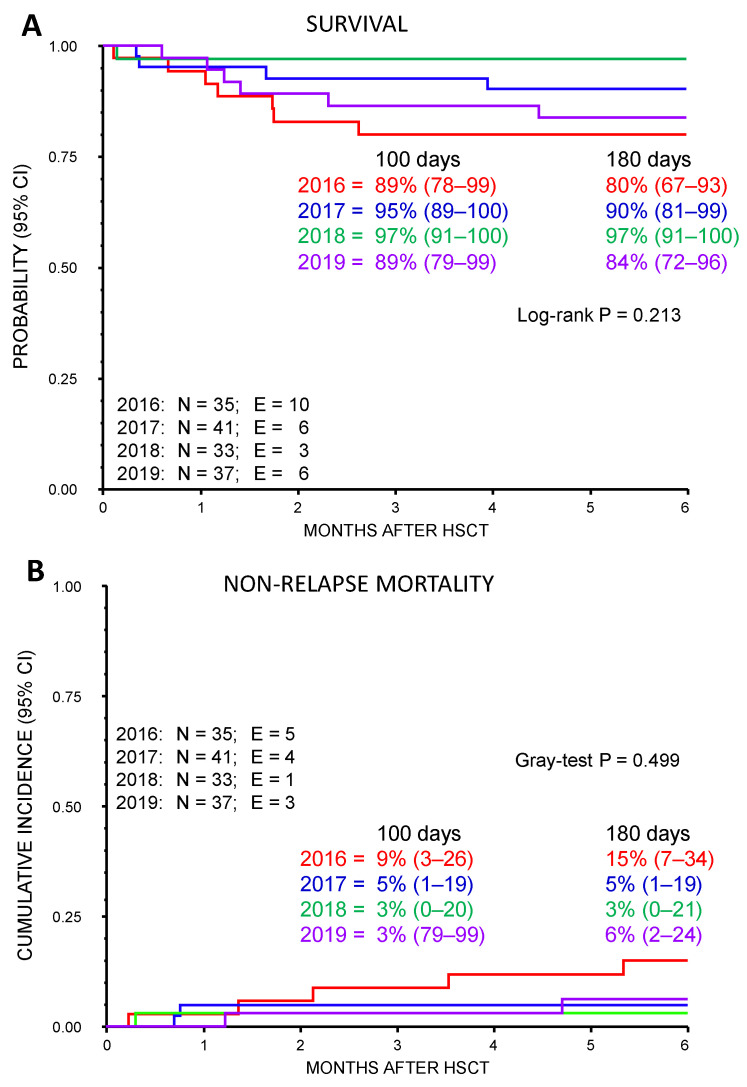
Survival probability (**A**) and non-relapse mortality (**B**) at 3 and 6 months after HSCT, according to the year of transplantation. Survival and non-relapse mortality were not statistically significantly different in 2016, 2017, 2018, and 2019, although there is a trend toward lower survival and higher non-relapse mortality for transplants performed in 2016.

Figure 1A,B show overall survival and non-relapse mortality by year of transplantation. The reduction of the transfusion threshold for GRC and PLT, implemented between 2016 and 2017, did not have a negative impact on patient outcomes. In fact, the difference in overall survival and non-relapse mortality according to the year of transplantation is not statistically significant and reflects the normal fluctuation of outcome over time.

## 4. Discussion

Our retrospective, single-center study, conducted in the inpatient and outpatient pediatric HSCT setting during the first 100 days post-transplant over a 4-year period, highlighted the safety (i.e., impact on adverse events and mortality) of progressively adopting a restrictive transfusion policy for both RBC and PLT. As depicted in Figure 1, the 1-year survival probability was comparable across the different years.

Our analysis shows that, in patients without hemorrhagic complications, the Hb transfusion threshold was already low as early as in 2016; nonetheless, we were able to further lower it (0.5 g/dL) since 2017 without decreasing patients’ safety; no patient died from hemorrhagic complications. In parallel, also the number of RBC units transfused decreased over time as well as the interval between the transfusion events. Despite the reduction in Hb threshold, we did not observe an increase in the number of patients with hemorrhagic complications; preliminary data on the patients analyzed seemingly do not show a difference in the Hb threshold over the years but rather in the number of transfusions administered.

We were also able to progressively decrease the PLT threshold in stable patients, reaching a threshold of 15,000/μL in 2019, despite having, as with Hb, a low former threshold. In 2018, the International Collaboration for Transfusion Medicine Guidelines (ICTMG) issued recommendations for platelet transfusion policy, and a platelet transfusion threshold of <10,000/μL was suggested for patients with hematologic malignancies and in the setting of HSCT [29]. However, in consideration of the higher risk of bleeding in the pediatric setting, our internal policy set the prophylactic PLT threshold for stable patients at 15,000/μL.

Similar to RBC transfusion, the total number of PLT units administered to our patients also decreased in 2019. The lower infusion of PLT and RBC led to a decrease in the transfusion-related risk, although we did not record a difference in the reported adverse events. This finding may be related to the high quality of the products administered. Of note, the transfusion threshold did not change in the outpatient cohort, for whom a more liberal prudential policy was usually adopted. Indeed, these patients underwent a strict monitoring of the blood count.

During the last decade, pediatric PBM programs were developed, mainly in the field of critical care [30,31,32], to reduce the risk of unnecessary exposure to blood products and to draft recommendations to optimize transfusion support and limit the use of the transfusion policy according to discretion of the attending physician. To date, few studies are available to investigate optimal transfusion management in HSCT patients, even though transfusion is a cornerstone of post-transplant support. Similar to the results obtained in critically ill adults [33], a few preliminary studies show that a restrictive RBC strategy may offer a clear advantage to the HSCT clinical setting. Indeed, a Canadian RCT trial conducted in 2013 in HSCT recipients, comparing an Hb threshold of 7 versus 12 g/dL, was stopped early due to the occurrence of severe veno-occlusive disease (VOD) in all patients enrolled in the 12 g/dL Hb threshold arm [34]. Likewise, a retrospective study conducted in 2011 [26] on HSCT children in stable conditions showed that a conservative RBC transfusion strategy (Hb 7 g/dL) was safe, as it did not affect clinical outcomes and mortality and lowered transfusion exposure. Another study demonstrated that a restrictive transfusion strategy in patients heavily treated before autologous HSCT for neuroblastoma was able to reduce the rate of mortality related to high levels of ferritin as a consequence of RBC administration [35]. Bleeding is a frequent complication in pediatric patients undergoing HSCT, mainly due to the myeloablative regimens that primarily induce prolonged aplasia and mucositis. The PLADO trial investigated the relationship between PLT dose and bleeding risk in adults and children. A subgroup analysis showed that bleeding risk was higher in children and inversely correlated with age [36]. Furthermore, the optimal transfusion threshold in stable patients has not been yet defined. Results from the investigations (mostly retrospective) conducted so far show that a prophylactic transfusion might be useful when PLT counts are between 10,000 and 20,000/μL [29]. Considering the lack of data, no definitive national and international guidelines are available to date for HSCT patients [28], thus the transfusion strategy is predominantly related to the single center policies or to the discretion of the attending physician [27]. As highlighted by the present study, the implementation of a restrictive policy by our group was not the consequence of the adoption of new standard operating procedures but rather depended on a change in the cultural sensitivity of treating physicians towards transfusion, due to the active collaboration with the transfusion service. Our approach was in contrast with the conviction of many transplant physicians who are often unwilling to change established transfusion thresholds as recently reported by Steffen and coworkers [37]. All our patients received a pre-transplant myeloablative-conditioning regimen, which implies a higher risk of developing complications such as mucositis, acute GvHD, and sepsis that may lead to a higher transfusion demand. The reduction in the number of units administered in both groups may be related to adherence to the PBM policy that suggests employing single units for each transfusion. If confirmed in a larger cohort, the adoption of a restrictive policy without impacting the patient’s safety may be important not only for the reduction of the transfusion-related risks but also for decreasing iron overload [17], especially in patients with a long transfusion history, such as those with sickle cell anemia or thalassemia, which represent more than 30% of our patients.

Well-designed studies are mandatory to clarify the threshold that allows for a benefit of prophylactic PLT transfusion in the HSCT setting, considering the alarm on PLT shortage in the face of increasing demand [38]. We are aware that our study has a number of limitations, mainly related to the retrospective design and to the relatively low number of patients. Nonetheless, it detected a change in the attitude of a homogeneous treating physicians’ team, as PBM awareness increased even without definite guidelines. The restrictive transfusion policy was demonstrated to be safe while also considering the peculiar context of pediatric HSCT patients and, not least, the doubts about maintaining an optimal Hb threshold in a growing organism. PBM is highly desirable in HSCT patients not only for offering the best transfusion policy but also because blood is a costly and increasingly rare resource due to the global aging population in the Western world.

Our preliminary study will guide the draft of internal guidelines for developing a new transfusion policy in the pediatric HSCT setting.

## 5. Conclusions

Transfusion should be considered a high-risk treatment, especially in children, due to its harmful potential. Well-designed prospective studies in pediatric patients undergoing HSCT should be mandatory for including this population in the PBM program.

## Figures and Tables

**Table 1:** Patient characteristics.

	N.	(% or Range)	(25th–75th Percentile)
Number of patients	146	(100%)	
Gender			
Male	83	(57%)	
Female	63	(43%)	
Median age at transplantation (years)	8	(0.6–18)	(4–12)
Median body weight at transplantation (kg)	24	(6–90)	(15–41)
Diagnosis			
Malignant disease			
Acute lymphoblastic leukemia	28	(19%)	
Acute myeloid leukemia	15	10%)	
Myelodysplasia	13	(9%)	
Hodgkin lymphoma	3	(2%)	
Non-Hodgkin lymphoma	7	(5%)	
Neuroblastoma	7	(5%)	
Ewing’s sarcoma	4	(3%)	
Non-malignant disease			
Bone marrow failure syndrome	14	(10%)	
Thalassaemia	25	(17%)	
Sickle cell anemia	25	(17%)	
Immunodeficiencies	5	(3%)	
Type of transplant			
Autologous	13	(9%)	
Matched family donor	27	(19%)	
Matched unrelated donor	50	(34%)	
Haploidentical	56	(38%)	
Conditioning regimen			
TBI-based	29	(20%)	
Busulfan-based	50	(34%)	
Treosulfan-based	46	(32%)	
Other chemotherapy	21	(14%)	
Stem cell source			
Bone marrow	64	(44%)	
Bone marrow + cord blood	6	(4%)	
Peripheral blood	76	(52%)	
Year of transplantation			
2016	35	(24%)	
2017	41	(28%)	
2018	33	(22%)	
2019	38	(26%)	

**Table 2:** Patient clinical outcome.

	Number of Patients	Events	100-Day Probability	(95% CI)	180-Day Probability	(95% CI)	1-Year Probability	(95% CI)
Survival	146	121	92%	(88–97)	89%	(84–94)	88%	(82–93
Event-fee survival	146	109	86%	(81–92)	82%	(76–88)	78%	(71–85)
Relapse	146	23	8%	(5–14)	10%	(6–17)	13%	(9–20)
Non-relapse mortality	146	14	5%	(2–10)	7%	(4–13)	8%	(5–14)
						N	(%)	
**Causes of death**								
Disease progression						11	(7%)	
Transplant-related causes						14	(10%)	
Acute GVHD						3		
Infectious complications						5		
Multi-organ failure						4		
Macrophage activation syndrome						1		
Liver veno-occlusive disease						1		
**Patients with hemorrhagic transplant complications**								
Total						8	(5%)	
Grade III-IV acute gastrointestinal GVHD						3		
Severe hemorrhagic cystitis due to Polyomavirus BK or Adenovirus infection						4		
Gastrointestinal thrombotic microangiopathy						1		

**Table 3:** Median pre-transfusion Hb level by year of transplantation and by occurrence of hemorrhagic complications. The reduction of the pre-transfusion Hb level was statistically significant (*p* = 0.010) in patients without major hemorrhagic complications, while no difference was observed in patients with hemorrhagic complications.

All Patients				
		Pre-Transfusion Hb Level (g/dL)		
Year	Number of Patients	Median	(Range)	(25th–75th Percentile)
2016	35	7.8	(5.1–10.0)	(7.5–8.0)
2017	41	7.3	(4.7–9.6)	(7.0–7.6)
2018	33	7.2	(5.3–10.9)	(7.0–7.5)
2019	38	7.2	(5.3–10.8)	(7.0–7.5)
*All Years*	*146*	*7.4*	*(4.7*–*10.9)*	*(7.1*–*7.6)*
**No Hemorrhagic Complications**				
		Pre-Transfusion Hb Level (g/dL)		
Year	Number of Patients	Median	(Range)	(25th–75th Percentile)
2016	31	7.8	(5.1–10.0)	(7.4–8.0)
2017	39	7.3	(4.7–9.6)	(6.9–7.5)
2018	33	7.2	(5.3–9.3)	(7.0–7.3)
2019	35	7.2	(6.0–10.8)	(7.0–7.5)
*All Years*	*138*	*7.3*	*(4.7*–*10.8)*	*(7.1*–*7.6)*
**Hemorrhagic Complications**				
		Pre-Transfusion Hb Level (g/dL)		
Year	Number of patients	Median	(Range)	(25th–75th percentile)
2016	4	8.0	(5.8–9.5)	(7.5–8.1)
2017	2	7.6	(5.6–8.9)	(7.0–7.9)
2018	0	- -	- -	- -
2019	2	8.0	(5.3–9.2)	(7.6–8.4)
*All Years*	*8*	*7.8*	*(5.3*–*9.5)*	*(7.3*–*8.0)*

**Table 4:** Median number of RBC transfusions given in the first 100 days after HSCT by year of transplantation and by occurrence of hemorrhagic complications. The median number of RBC transfusions per patients remained unchanged over time.

All Patients				
		Number of RBC Transfusions in the First 100 Days		
Year	Number of Patients	Median	(Range)	(25th–75th Percentile)
2016	35	3	(0–31)	(2–6)
2017	41	4	(0–82)	(2–7)
2018	33	3	(0–10)	(2–5)
2019	38	3	(0–18)	(1–6)
*All Years*	*146*	*3*	*(0*–*82)*	*(2*–*6)*
**No Hemorrhagic Complications**				
		Number of RBC transfusions in the First 100 Days		
Year	Number of Patients	Median	(Range)	(25th–75th Percentile)
2016	31	3	(0–12)	(1–5)
2017	39	3	(0–12)	(2–6)
2018	33	3	(0–10)	(2–5)
2019	35	2	(0–12)	(1–4)
*All Years*	*138*	*3*	*(0*–*12)*	*(2*–*5)*
**Hemorrhagic Complications**				
		Number of RBC Transfusions in the First 100 days		
Year	Number of Patients	Median	(Range)	(25th–75th Percentile)
2016	4	23	(13–31)	(14–31)
2017	2	50	(18–82)	(18–82)
2018	0	- -	- -	- -
2019	2	17	(16–18)	(16–18)
*All Years*	*8*	*18*	*(13*–*82)*	*(15*–*31)*

**Table 5:** Median pre-transfusion level by year of transplantation and by occurrence of hemorrhagic complications. The pre-transfusion count decreased significantly in children without hemorrhagic complications (*p* = 0.026), while it remained higher and unchanged in those with hemorrhagic complications.

All Patients				
		Pre-Transfusion PLT Level (×10^3^/μL)		
Year	Number of Patients	Median	(Range)	(25th–75th Percentile)
2016	35	18	(3–50)	(16–19)
2017	41	16	(1–32)	(14–18)
2018	33	15	(2–33)	(14–18)
2019	38	15	(4–49)	(14–18)
*All Years*	*146*	*15*	*(1*–*50)*	*(14*–*18)*
**No Hemorrhagic Complications**				
		Pre-Transfusion PLT Level (×10^3^/μL)		
Year	Number of patients	Median	(Range)	(25th–75th Percentile)
2016	31	18	(12–40)	(16–19)
2017	39	16	(10–32)	(13–18)
2018	33	15	(11–33)	(14–18)
2019	35	15	(12–49)	(14–18)
*All Years*	*138*	15	(10–49)	(14–18)
**Hemorrhagic Complications**				
		Pre-Transfusion PLT Level (×10^3^/μL)		
Year	Number of Patients	Median	(Range)	(25th–75th Percentile)
2016	4	15	(3–50)	(10–20)
2017	2	23	(3–32)	(17–26)
2018	0	- -	- -	- -
2019	2	18	(4–42)	(14–24)
*All Years*	*8*	*16*	*(3*–*50)*	*(13*–*21)*

**Table 6:** Median number of PLT transfusions given in the first 100 days after HSCT by year of transplantation and by occurrence of hemorrhagic complications. It remained low throughout the whole study period.

All Patients				
		Number of PLT Transfusions in the First 100 Days		
Year	Number of Patients	Median	(Range)	(25th–75th Percentile)
2016	35	6	(0–63)	(2–11)
2017	41	7	(0–50)	(4–15)
2018	33	7	(0–17)	(4–10)
2019	38	5	(0–39	(3–11)
*All Years*	*146*	*6*	*(0*–*63)*	*(3*–*11)*
**No Hemorrhagic Complications**				
		Number of PLT Transfusions in the First 100 Days		
Year	Number of Patients	Median	(Range)	(25th–75th Percentile)
2016	31	5	(0–22)	(2–9)
2017	39	7	(0–33)	(4–13)
2018	33	7	(0–17)	(4–10)
2019	35	4	(0–18)	(3–11)
*All Years*	*138*	6	(0–33)	(3–10)
**Hemorrhagic Complications**				
		Number of PLT Transfusions in the First 100 Days		
Year	Number of Patients	Median	(Range)	(25th–75th Percentile)
2016	4	35	(11–63)	(16–57)
2017	2	35	(20–50)	(20–50)
2018	0	- -	- -	- -
2019	2	26	(13–39)	(13–39)
*All Years*	*8*	*35*	*(11*–*63)*	*(15*–*47)*

## Data Availability

The data presented in this study are available on request from the corresponding author. The data are confidential.
